# Plant Polyphenols and Their Potential Benefits on Cardiovascular Health: A Review

**DOI:** 10.3390/molecules28176403

**Published:** 2023-09-01

**Authors:** Iram Iqbal, Polrat Wilairatana, Fatima Saqib, Bushra Nasir, Muqeet Wahid, Muhammad Farhaj Latif, Ahmar Iqbal, Rabia Naz, Mohammad S. Mubarak

**Affiliations:** 1 Department of Pharmacology, Faculty of Pharmacy, Bahauddin Zakariya University, Multan 60800, Pakistan; iramiqbal.bzu@gmail.com (I.I.); muqeetsoomro@msn.com (M.W.); farhajlatif@gmail.com (M.F.L.); rabianazbhutta@gmail.com (R.N.); 2 Department of Clinical Tropical Medicine, Faculty of Tropical Medicine, Mahidol University, Bangkok 10400, Thailand; fatima.saqib@bzu.edu.pk; 3 Department of Pharmaceutics, Faculty of Pharmacy, Bahauddin Zakariya University, Multan 60800, Pakistan; bushranasir@bzu.edu.pk; 4 Department of General Surgery, Shanxi Medical University, Jinzhong 030600, China; ahmar204@yahoo.com; 5 Department of Chemistry, The University of Jordan, Amman 11942, Jordan

**Keywords:** polyphenols, cardiovascular, atherosclerosis, oxidative stress

## Abstract

Fruits, vegetables, and other food items contain phytochemicals or secondary metabolites which may be considered non-essential nutrients but have medicinal importance. These dietary phytochemicals exhibit chemopreventive and therapeutic effects against numerous diseases. Polyphenols are secondary metabolites found in vegetables, fruits, and grains. These compounds exhibit several health benefits such as immune modulators, vasodilators, and antioxidants. This review focuses on recent studies on using dietary polyphenols to treat cardiovascular disorders, atherosclerosis, and vascular endothelium deficits. We focus on exploring the safety of highly effective polyphenols to ensure their maximum impact on cardiac abnormalities and discuss recent epidemiological evidence and intervention trials related to these properties. Kaempferol, quercetin, and resveratrol prevent oxidative stress by regulating proteins that induce oxidation in heart tissues. In addition, polyphenols modulate the tone of the endothelium of vessels by releasing nitric oxide (NO) and reducing low-density lipoprotein (LDL) oxidation to prevent atherosclerosis. In cardiomyocytes, polyphenols suppress the expression of inflammatory markers and inhibit the production of inflammation markers to exert an anti-inflammatory response. Consequently, heart diseases such as strokes, hypertension, heart failure, and ischemic heart disease could be prevented by dietary polyphenols.

## 1. Introduction

Cardiovascular disease (CVD), encompassing conditions such as atherosclerosis, hypertension, myocardial infarction, cardiomyopathy, arrhythmia, and heart failure (HF), is a major contributor to global mortality. The incidence of CVD has experienced a notable increase [1,2,3,4]. Despite the wide range of pharmaceuticals currently utilized for the management of CVD, such as statins, angiotensin-converting enzyme inhibitors (ACEIs), angiotensin receptor blockers (ARBs), calcium channel blockers (CCBs), fibrates, and β-blockers, it is important to acknowledge that a significant number of these medications are associated with adverse effects in the human population [4]. Hence, there exists a significant clinical requirement to discover and cultivate innovative therapeutic strategies for CVD [2]. The transport of oxygen and nutrients in the human body is carried out via blood circulation along with the removal of metabolic by-products and carbon dioxide through the cardiovascular system (CVS). Coronary artery disease (CAD), cerebrovascular disease (CVD), peripheral artery disease (PAD), congenital heart disease (CHD), hypertension, heart failure, and stroke are all disorders that affect the heart and blood arteries [1,2]. Within this context, cardiovascular diseases (CVDs) are among the leading causes of mortality throughout the world, claiming 17.9 million individual lives worldwide in 2019, which is approximately 32% of total fatalities. Approximately, 85% of these mortality rates were due to heart attacks and strokes (WHO site). Strokes kill 6.7 million people each year, and coronary heart disease claims 7.4 million lives [3,4].

Several pathologies can affect the cardiovascular system. Some of these pathologies include primary heart ailments, including cardiomyopathy and cardiac malignancies. Infectious and infectious-allergic damage to heart tissue, metabolic and systemic disorders, and diseases of other organs are also covered in this category [5,6]. CHD starts with inflammation of the blood artery walls, which narrows and causes angina pectoris [7]. In this respect, blood clots restrict arteries later in the disease’s progression, resulting in severe myocardial ischemia and myocardial infarction (heart attack). Heart failure can occur in severe cases of CHD when the heart muscle’s ability to pump blood around the body deteriorates [2]. Because these disorders are generally caused by arterial injury, symptoms and treatments vary depending on which arteries are afflicted [2].

Age and gender are among the most reported non-modifiable cardiovascular risk factors. Cardiovascular disorders become more common as people become older due to a rise in plasma cholesterol on one hand and the augmentation of arterial rigidity and peripheral vascular resistance on the other hand [8]. Although the risk of cardiovascular disorder varies with gender and age, the incidence is three to five times greater in men < 50 years of age compared to women. On the other hand, a considerable increase in the occurrence of CVD has been observed in women over the age of 50 years. Genetic factors, inactivity, hypertension, obesity, diabetes, smoking, and dyslipidemia are the prominent risk factors for cardiovascular disorders, as described in published reports [9,10,11,12]. 

Research findings have shown that a balanced diet is beneficial for preventing CVD [13]. The consumption of a high percentage of fruits and vegetables in a diet, such as the Vegan diet, is strongly correlated with a long life expectancy. In addition, it decreases the incidence of cardiovascular diseases [14]. According to epidemiological research, people who consume a diet rich in polyphenols experience a 46 percent reduction in their risk of developing CVD [15]; these food items are rich in polyphenols. Polyphenols are present in vegetables and in many fruits and seeds that we routinely consume as secondary metabolites [16]. In this regard, there is a connection between the consumption of fruits, vegetables, seeds, and nuts and a decreased incidence of chronic and age-related degenerative illnesses [17].

**Figure 1:**
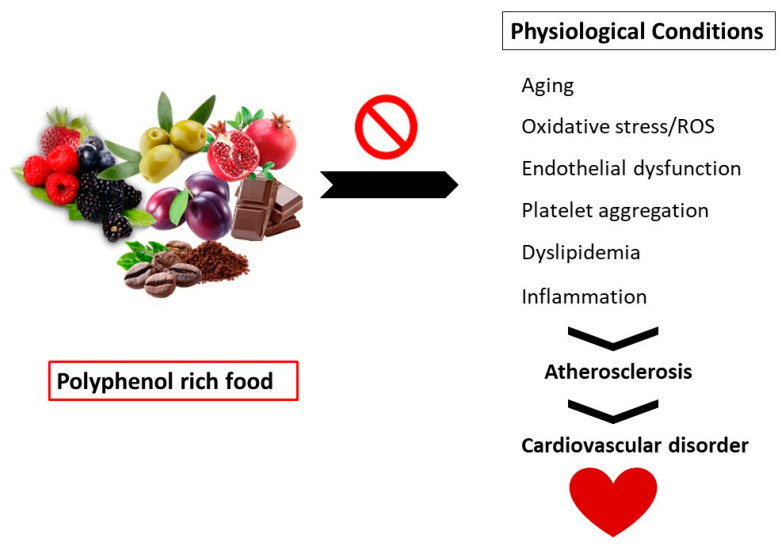
Nutrition can help prevent atherosclerosis, which is a pathophysiological process that contributes to the development of cardiovascular disease (CVD).

Polyphenols have also been found useful in enhancing endothelial function, preventing aberrant platelet aggregation, decreasing inflammation, and improving plasma lipid profile, all of which benefit cardiovascular health. Because the processes by which these chemicals exert cardioprotective activities are not entirely known, although not conclusively shown, there may be a connection between the cardiovascular advantages of some diets and their polyphenol levels [18]. Based on the preceding remarks, this work focuses on summarizing the literature dealing with the pharmacological effects of dietary polyphenols and presents an overview of the recent developments regarding their use in the prevention and treatment of different diseases. We hope that this paper can be beneficial to future research and the development of new therapeutic strategies. Depicted in Figure 1 is a sketch that shows how nutrition can help in preventing atherosclerosis, which contributes to CVD. 

## 2. Polyphenols

Polyphenols are phytochemicals or secondary plant compounds that are considered non-essential nutrients in plants [19]. They are a rich collection of chemicals present in plants and algae, where their natural role is to defend the organism against UV radiation, infection, and herbivore ingestion. Polyphenols come in a variety of structural forms, from basic monomers to complex polymerized structures. Seaweed polyphenols may help lower hyperglycemia, hyperlipidemia, oxidative stress, chronic inflammation, metabolic abnormalities linked to CVDs, and diabetes sequelae. On the other hand, polyphenols from plants have been related to improved health in terms of obesity, diabetes, and CVD. A recent study has focused on marine macroalgae, presumably because of epidemiological evidence from Asian nations that suggests a diet high in seaweed lowers the occurrence of CVD, cancer, and other chronic disorders [1,20].

**Figure 2:**
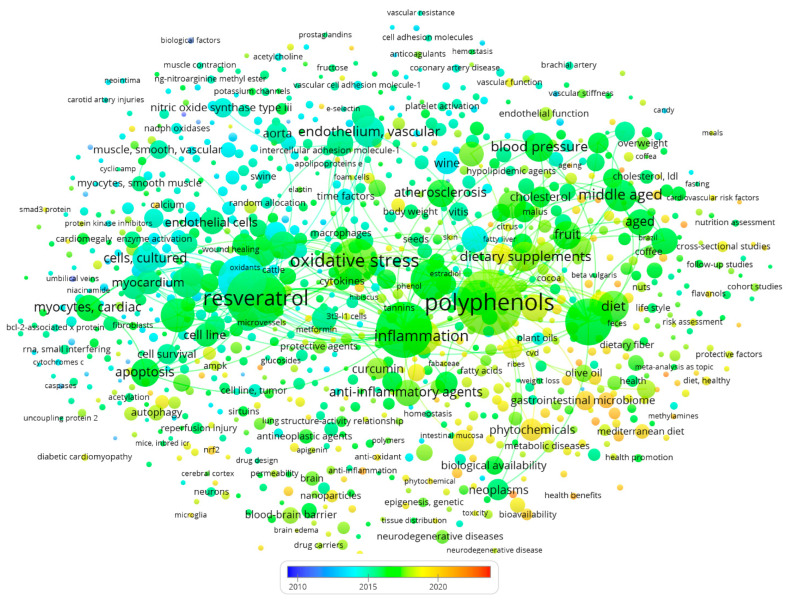
Scientific Literature analysis for the effect of polyphenols on cardiovascular diseases. This bibliography data were extracted from PubMed with the year range set to 2010–2023.

Polyphenols are also outstanding plant-derived secondary metabolites that exhibit anticancer, anti-cardiovascular, antidiabetic, and anti-neurodegenerative properties. Compounds like phenicic acid, stilbenes, flavonoids, coumarins, tannins, and lignins are present in numerous plants, including tonka bean (*Dipteryx odorata*), sweet grass (*Hierochloe odorata*), sweet woodruff (*Galium odoratum*), deer-tongue grass (*Dichanthelium clandestinum*), sweet clover (*Verbascum* spp.), and vanilla grass (*Anthoxanthum odoratum*). In addition to its antioxidant characteristics, resveratrol also exerts ameliorating effects against inflammation, cancer, aging, obesity, and diabetes, along with cardioprotective and neurological benefits [21]. A scientific literature analysis on PubMed with the keywords “Cardiovascular Polyphenols” showed that around 4,000 papers have been published between 2010 and 2023. Figure 2 represents our keyword occurrence analysis, in which the most focused research keywords were oxidative stress, inflammation, resveratrol, atherosclerosis, endothelium, dietary supplement, blood pressure, and apoptosis.

Phenolic compounds are the most copious non-energetic components in plant-based meals. The aptitude of polyphenols to alter enzymatic activity and, consequently, the signal-transmitting mechanisms of several processes occurring in cells may be attributed to their physicochemical properties, which allow polyphenols to participate in numerous metabolic cellular redox processes. Thus, the antioxidant scavenging properties of polyphenols make them advantageous. These are the most predominant antioxidants in the diet; they are 20 times more prevalent than vitamin E and carotenoids and 10 times more prevalent than vitamin C. Nicotinamide-adenine-dinucleotide phosphate (NADP) oxidase and xanthine oxidase are two ROS-producing enzymes that polyphenols can inhibit [22]. Listed in Table 1 are polyphenol-rich plant foods.

## 3. Classification of Polyphenols

**Figure 3:**
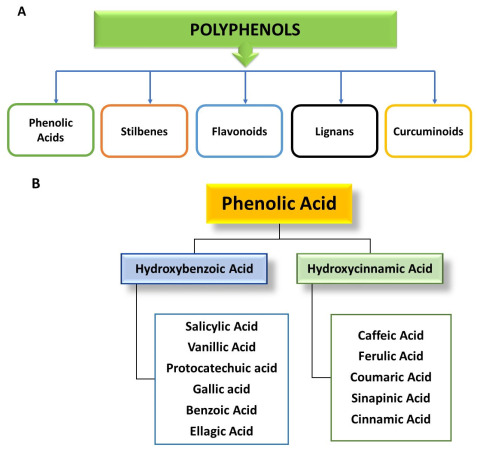
(**A**) Classification of polyphenols. (**B**) Classification and food sources of phenolic acids.

Polyphenols are divided into several categories, including phenolic acids (hydroxybenzoic and hydroxycinnamic acids), flavonoids (flavones, flavonols, isoflavones, flavanones, and anthocyanins), stilbenes (resveratrol, piceatannol), lignans (sesamol, pinoresinol, sinol, enterodiol), and others, including tannins (hydrolyzable, non-hydrolyzable, and condensed tannins), lignins, xanthones, chromones, and anthraquinones, as shown in Figure 3A [28,29,30].

### 3.1. Phenolic Acids

Phenolic acids are a class of organic compounds composed of aromatic rings connected to a carboxylic acid group. The antioxidant properties of phenolic acids make them protective against CVD [28,30,31]. Vegetarian food, including seeds, fruits, and green-colored vegetables, are known to be good sources of phenolic acids. In addition to their health-promoting benefits, phenolic acids are widely used in a variety of things, including cosmetics, food, and medicine [32]. Phenolic acids are divided into hydroxybenzoic and hydroxycinnamic acids. 

#### 3.1.1. Hydroxybenzoic Acids

Hydroxybenzoic acids are benzoic acid (C_7_H_6_O_2_) derivatives. The Hydroxybenzoic acids sub-category includes salicylic acid, protocatechuic acid, vanillic acid, benzoic acid, gallic acid, and ellagic acid [30,33]; gallic acid may be found in large quantities in tea and grape seeds [34]. Included within olive products are hydroxybenzoic acids, which exert anti-inflammatory, antioxidant, and cardioprotective properties, among others [29,30,31,35]. 

#### 3.1.2. Hydroxycinnamic Acids

Aromatic acids, which are derived from cinnamic acid are represented by the family of hydroxycinnamic acids (C6-C3) [29,36]. Adequate sources of hydroxycinnamic acids include coffee, berries, apples, grains, and kiwi fruit [37,38]. Specifically, coffee contains chlorogenic acid, and caffeine, berries, and apples contain caffeic acid, while cereals contain ferulic acid. Most citrus fruits contain caffeine and chlorogenic acid (cinnamic acid) [34]. In addition to having anti-inflammatory properties, phenolic acids can also protect the body from cell damage, ROS, oxidative stress, and cardiovascular health issues like heart diseases and diabetes; they have neuroprotective and food-preservative properties that can help keep food fresh longer [30,34]. Figure 3B depicts the classification of phenolic acids.

### 3.2. Lignin

Lignin is a category of complex chemical compounds that may be found in a variety of plant tissues. Lignin is particularly important in plants and trees because it assists in cell wall formation [39]. Flaxseeds, tomatoes, peaches, apples, and some berries are examples of foods high in lignin [30].

#### Silymarin

Lignin silymarin, a kind of flavonolignan, has been shown to possess antioxidant properties. This kind of lignin may be found in the seeds of milk thistle and other herbaceous plants [40].

### 3.3. Stilbenes

Stilbenes are phenol-derived metabolites (C_14_H_12_). Their biological activity and health-promoting benefits are a source of research interest and a focal point for many studies. Studies have focused on their bioavailability, metabolism, and absorption rates, as well as their overall health benefits [41].

#### Resveratrol

The most well-known kind of stilbene is resveratrol, which has anti-inflammatory effects [42]. Resveratrol is mostly found in grapes and red wine. This compound has also been demonstrated to lower blood pressure. Additionally, studies have indicated that taking resveratrol as a supplement rather than getting it naturally is more beneficial. Moreover, findings have indicated that resveratrol may be beneficial to humans, although its specific mechanism of action is currently being researched [43]. Despite its limited bioavailability in the body, resveratrol has been shown to protect against CVDs and have a sun-protective impact, thus protecting against skin cancer [44].

### 3.4. Flavonoids

**Figure 4:**
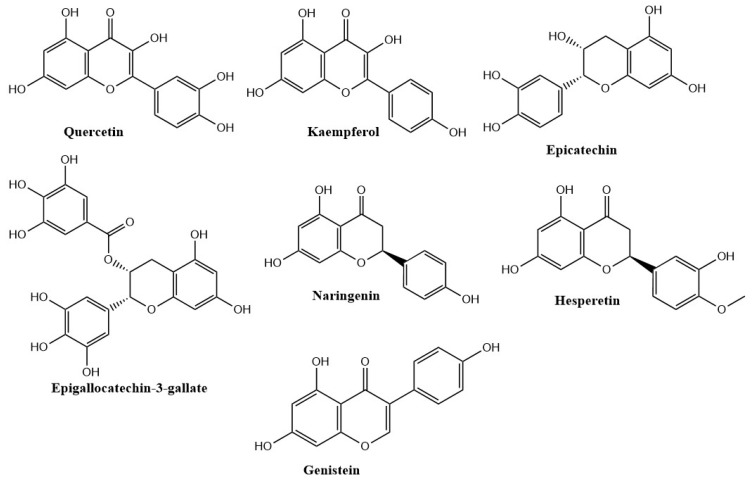
Chemical structures of important phytocompounds.

Flavonoids are naturally occurring polyphenolic compounds that are divided into six primary categories: flavanones, flavones, flavanols, isoflavones, flavan-3-ols, and anthocyanidins [44]. Figure 4 depicts the chemical structures of important flavonoids.

#### 3.4.1. Flavones

Flavones are present in foods like garlic, chamomile tea, and celery, which are rich in luteolin [45]. The advantageous effects of luteolin that have been observed in various studies include blood pressure reduction in hypertensive rats, improving vasodilation of the aortic rings, and increasing cAMP accumulation due to the inhibition of cAMP-specific phosphodiesterase [46]. The activation of the cAMP/PKA cascade increases endothelial cell nitric oxide levels through the activation of endothelial enzyme nitric oxide synthase. This encourages the relaxation of the vasculature through nitric oxide, a mechanism carried out by potassium and calcium channels [47]. 

#### 3.4.2. Flavonols

Onions, tea, broccoli, and fruit are rich sources of flavonols, which are represented by kaempferol and quercetin, which are glycosides [48]. 

##### Quercetin

Quercetin exerts its antihypertensive effect by improving endothelial function, modulating the renin–angiotensin–aldosterone system (RAAS) by modulating the mechanism of contraction of smooth muscles in blood vessels [48], producing vasodilation at a renal level that is protein kinase C-dependent, and lowering blood pressure in patients with diabetes or metabolic syndrome [49,50]. Quercetin is reported to cause oxidative stress reduction in the heart and kidneys [51].

##### Kaempferol

Kaempferol is present in foods such as broccoli, strawberries, green tea, and beans [52]; its antihypertensive effects are manifested by the activation of endothelial nitric oxide [53]. Besides its antihypertensive effect, kaempferol is reported to reduce proteinuria and albuminuria, and it is considered a potential contender for the improvement of these two conditions [54]. 

#### 3.4.3. Flavan-3-ols

Flavan-3-ols comprise monomers such as epicatechin, gallocatechin, catechin, and oligomers (proanthocyanidin) [52]. Catechin monomers are found in apple-, tea-, cocoa-, pear-, and grape-based products in the form of aglycones (part of non-carbohydrate glycosides). Catechins have been shown to have advantageous effects on vascular function and to be cardioprotective. Additionally, studies have revealed that they can lower both systolic and diastolic blood pressure [55,56]. 

##### Epicatechin

Epicatechin-rich food results in decreasing both systolic blood and diastolic blood pressure by 4.2 mmHg and 2.1 mmHg, respectively, showing its antihypertensive effect. It has also been found to decrease myocardial rigidity in hypertrophic cardiomyopathic rats [57]. 

##### Epigallocatechin-3-gallate

Epigallocatechin-3-gallate is abundant in green tea and has been found to have antioxidant, anti-inflammatory, and antiatherogenic properties [58]. 

#### 3.4.4. Flavanones

Naringenin and hesperetin are the main representatives of this class, which is predominantly found in citrus fruits, especially in their peels [59]. Their antioxidant properties are due to their free radical scavenging activity [60].

##### Naringenin

Naringenin has some promising effects; e.g., it reduces mean blood pressure, regulates nitric oxide levels, and provides a shield against endothelial dysfunction [61,62]. 

##### Hesperetin

Hesperetin is one of the dietary flavanones found in citrus fruits [52] which is rapidly absorbed in the intestine, and the resulting metabolites are responsible for the antihypertensive effect. They can also reduce the progression of atheroma plaque through their anti-inflammatory activity. In addition to this, the antioxidant effect of hesperetin helps to increase the amount of nitric oxide and reduces the amount of calcium ions, thus producing smooth muscle relaxation in the blood vessels [63,64].

#### 3.4.5. Anthocyanidins

Anthocyanidins are key soluble pigments that provide color to fruits and vegetables like blue, red, or purple fruits, e.g., forest fruits and black currants, etc. [52]. The endothelium-dependent vasodilatory property has beneficial effects on the cardiovascular system, thereby reducing the risk of acute myocardial infarction [65].

#### 3.4.6. Isoflavones

Soy is a source of isoflavones, which are structurally similar to mammalian estrogens. Thus, they can produce their agonistic effect by binding to estrogen receptor agonists. Diadzein and genistein are the two main isoflavones in this class [66]. 

##### Diadzein

Diadzein has been found to have an anti-damaging effect by reducing oxidative stress, increasing nitric oxide synthesis, reducing LDL oxidation, and increasing prostaglandin production [67]. 

##### Genistein

Genistein has the property of reducing hypertension [68].

## 4. Bioavailability of Polyphenols

The bioavailability of phenolic compounds in our food is critical because only the most bioavailable phenolic compounds in our diet will have the most beneficial effects on the human body [69]. These will be different for each person depending on their relationship with food, how cell walls are made, and where glycosides are found. Many epidemiological studies have shown that phenolic compounds have a lot of health benefits, such as protecting against the buildup of fat, preventing microorganisms from decaying, lowering cardiovascular diseases, preventing diabetes, stroke, and cancer, and exerting anti-inflammatory effects [30].

**Figure 5:**
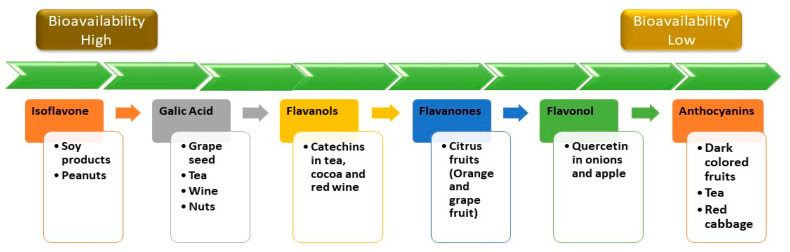
Comparative bioavailability of some common dietary polyphenols.

Recent research has placed a strong emphasis on identifying the processes governing polyphenol metabolism and bioavailability in humans [70]. A wide array of fruits and vegetables contain compounds known as phenolics. Some plants contain as much as 750 mg/100 g of fruit, which is a significant amount [19,71]. The highest dietary sources of polyphenols are dark-colored fruits (especially small berries), chocolate, cereals made entirely of whole grains, coffee, and red wine, with the latter three accounting for the lion’s share of overall dietary polyphenol consumption [72]. Among the food groups consumed, polyphenols are primarily associated with carbohydrates, organic acids, and other food groups. They combine with arabinose to generate ester linkages in hemicellulose or core lignin, which allows them to form covalent connections with polysaccharides in the cell wall of the plant. While flavonoids can be found in the cytosol and endoplasmic reticulum, where they are formed, they are mostly found in free form in the cytosol and endoplasmic reticulum. Cell barriers and intracellular compartments must be damaged for the drug to be bioavailable [71]. Flavonoids found in nature are housed within plants as glycoside and non-glycosylated conjugate compounds, and as a result, the moiety’s type might affect their subsequent human bioavailability [73,74]. A summary of the comparative bioavailability of different polyphenols is shown in Figure 5 [75].

### 4.1. Metabolism of Polyphenols

#### 4.1.1. Oral and Gastric Absorption

The most prevalent enzyme in the mouth cavity is amylase, which starts the digesting process. Due to the brief contact duration, the consequence of enzyme action on polyphenol release from food is predicted to be negligible [76]. On the other hand, particle size decrease occurs, resulting in particles with a diameter ranging from a few hundred microns to several thousand microns [77], allowing for increased enzyme access throughout the succeeding stages of digestion due to an increase in the digest volume fraction. Most polyphenols seem to be formed in the stomach during the digestion process. In the gastric phase digestion with pepsin, peristaltic movements, and a low pH cause formation of finely powdered digestible polyphenols with even smaller particle sizes, often less than 500 microns in diameter [78]. Another factor that might contribute to polyphenols remaining in an undissociated form is the low pH, which may accelerate the movement of polyphenols from the food matrix to the aqueous phase due to decreased interactions between ionic groups. The pH of the digestive fluids normally rises from approximately 2–4 to around 7 when digested food exits the stomach and enters the small intestine. It is possible that the pancreatic and biliary enzymes can be activated in this manner [79]. A brush border enzyme called lactase-phlorizin hydrolase (LPH) is thought to be responsible for cleaving polyphenols from their sugar moiety before cellular absorption [80].

#### 4.1.2. Uptake in Enterocytes

The gastric absorption of some polyphenols has been strongly suggested because of how soon these polyphenols enter plasma after consumption [81]. After breaking into their corresponding aglycons, polyphenols can penetrate the intestinal epithelium via passive transport, active transport, or facilitated transport. Polyphenols with low molecular weight, like phenolic acids, flavonoid aglycon, herbal tea polyphenols, and the polyphenols in cocoa (epicatechin, procyanidin B2, catechin), are believed to be absorbed mostly by passive diffusion (based on tests in Caco-2 cells) [82,83,84]. 

The hypothesis that sodium-glucose transport, notably the protein known as “sodium-glucose-linked transporter 1 (SGLT1)”, actively takes up specific glycoside polyphenols has been proposed [85]. According to these findings, glycosides may be absorbed by SGLT1 to a small amount before being re-secreted into the digestive system, or they may be further broken down by cytosolic glucosidase in the body [86]. Another approach for polyphenol absorption is the use of monocarboxylic acid transporters to facilitate the transfer of polyphenolic substances into enterocytes (MCTs). To be recognized as an absorption substrate, a polyphenol must contain an attached carboxylic acid group of a monoanionic nature as well as a side chain with a nonpolar nature or an aromatic group with a hydrophobic nature [87,88]. MCTs have been shown to take up a range of polyphenols, such as caffeic and ferulic acid (usually utilizing Caco-2 cell models) [89]. 

#### 4.1.3. Effect of Microbial Fermentation in the Colon

While some nutritional flavonoids are taken in the small intestine, the majority are taken in the intestinal tract, where the intestinal microbiota additionally degrades the deconjugated type metabolites and related compounds into freely accessible compounds such as phenolic acids, which are subsequently absorbed [90]. Bacteria may have an essential role in the breakdown of plant polyphenols and phase I/II metabolism conjugates, which are usually discharged by enterohepatic recirculation. Glycosylation, hydroxylation, demethylation, deconjugation, ring cleavage (typical of the C-ring), hydrolysis, epimerization, and chain-shortening processes are among the routine occurrences [88,91]. 

#### 4.1.4. Metabolism in the Enterocytes

Most polyphenols are supposed to be absorbed in the small intestine, where they are commonly processed by phase II enzymes before they enter systemic circulation [74]. After they have been extensively captivated by the intestinal epithelial cells, before inflowing into the systemic flow, they are subjected to phase II enzymatic detoxification, which results in the creation of various conjugated harvests. These include sulfates produced by the action of sulfotransferases (SULTs), glucuronides produced by the action of uridine-5′-diphosphate glucuronosyltransferases (UGT), and some methylated derivatives produced by catechol-O-methyltransferase (COMTs) action [19,92]. Aspects of polyphenol bioavailability and accumulation in tissues are also strongly related to the action of some proteins, mainly “multidrug resistance-associated proteins” such as MRP-1/MRP-2. These are efflux transporters that are ATP-dependent for their action, and together, they are referred to as phase III metabolism [93]. Aglycones undergo further phase II metabolism after being oxidatively degraded in hepatocytes, particularly in the Golgi apparatus and the peroxisome [92,94].

#### 4.1.5. Distribution in Body and Excretion

Polyphenolics can be carried in the bloodstream in three different ways: (1) in free form, (2) when coupled to proteins, and (3) when lipoprotein (lipids)-bound. Polyphenols appear to be coupled to proteins in most cases [95,96]. In the end, via the portal blood flow, they make their way into the liver, where they move through a second phase of metabolism (phase II) before entering the systemic blood flow and peripheral bodily tissues, where they are eliminated by the kidneys [97].

**Figure 6:**
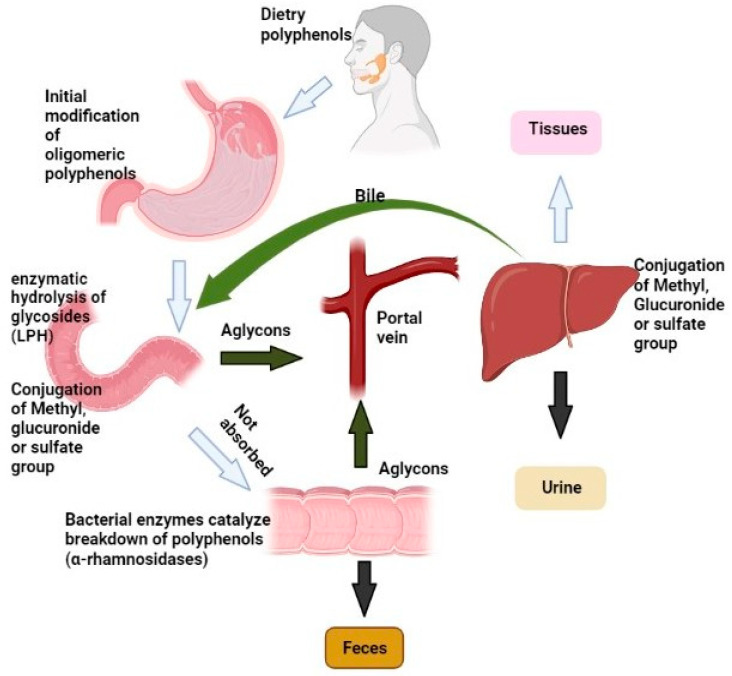
A schematic diagram of the route of absorption, metabolism, and excretion of polyphenols.

Polyphenols are believed to mostly be excreted through urinary excretion, especially those with greater hydrophilicity. The percentage of polyphenols retrieved in urination (including conjugates) varies greatly, with gallic acid and isoflavones having the largest amounts (over 60%) of polyphenols recovered [19,98,99]. Figure 6 shows a schematic diagram of the route of absorption, metabolism, and excretion of polyphenols, and Table 2 shows a summary of the most prevalent dietary polyphenols and their major colonic metabolites.

## 5. Role of Vascular Endothelium in the Regulation of Vascular Homeostasis

A monolayer of cells produces the endothelium, which makes up the interior of blood vessels. Vascular endothelium regulates the tone and homeostasis of the vasculature, as well as the morphological changes that occur in pathological circumstances. The endothelium regulates the balance of opposing processes such as vasodilation and constriction, pro-coagulant and antithrombotic actions, and cell proliferation and apoptosis [115,116].

**Figure 7:**
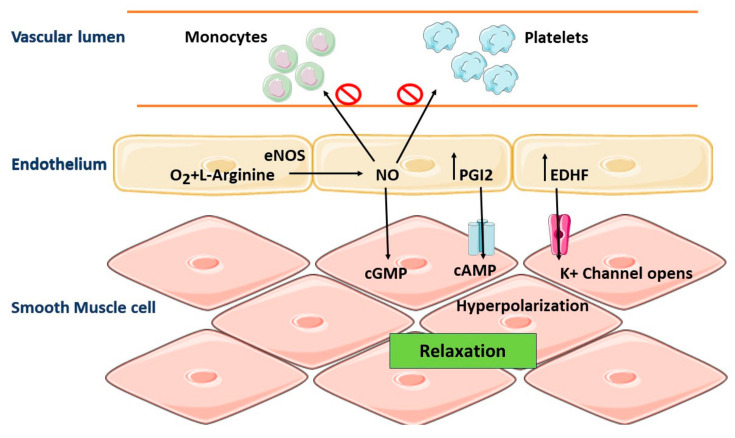
**Normal regulation of vascular homeostasis**. Vascular homeostasis is regulated in part by endothelium-derived NO. The endothelial NO synthase (eNOS) enzyme produces NO from L-arginine, exerting a vasodilatory effect. NO can easily diffuse into the cells of vascular smooth muscle, where it triggers guanyl cyclase, thus accumulating cyclic guanosine monophosphate (cGMP), which ultimately activates the protein kinase G and causes vasorelaxation in endothelial.

Endothelial cells minimize the interaction of the bloodstream with the basal prothrombotic arterial wall due to their selective position [117]. The primary function of Endothelial cells is to regulate vascular tone by producing vasodilator and vasoconstrictor chemicals. The endothelial NO synthase (eNOS) enzyme produces NO from L-arginine, exerting a vasodilatory effect. NO can easily diffuse into the cells of vascular smooth muscle, where it triggers guanyl cyclase, thus accumulating cyclic guanosine monophosphate (cGMP), which ultimately activates the protein kinase G and causes endothelial vasorelaxation (Figure 7). The endothelium-derived hyperpolarizing factor (EDHF) plays its role in vasodilation by targeting the K+ channels in the blood vessels. Furthermore, prostacyclin (PGI2) produced during the cyclooxygenase (COX) pathway has vasodilatory effects. Some other factors can be produced by the endothelium having vasoconstrictive effects on blood vessels such as angiotensin II (Ang II), endothelin-1 (ET-1), and thromboxane A2 (TXA2) [15].

Regarding blood–tissue contact, the endothelium does play a crucial role, interacting directly with a variety of circulating substances, including antioxidants, oxidized LDLs, and pro-inflammatory cytokines such as tumor necrosis factor (TNF) and interleukins (IL) [115,116]. These variables can cause vasomotricity or the manufacturing of endothelial agents like nitric oxide (NO). Their role in a variety of physiological processes has been reported to influence biological processes such as the apoptosis, proliferation, and migration of endothelial cells [15,118,119]. Thus, endothelial dysfunction can have several detrimental effects on vascular cells and surrounding tissue, resulting in the development of cardiovascular disorders such as atherosclerosis and hypertension [120].

**Figure 8:**
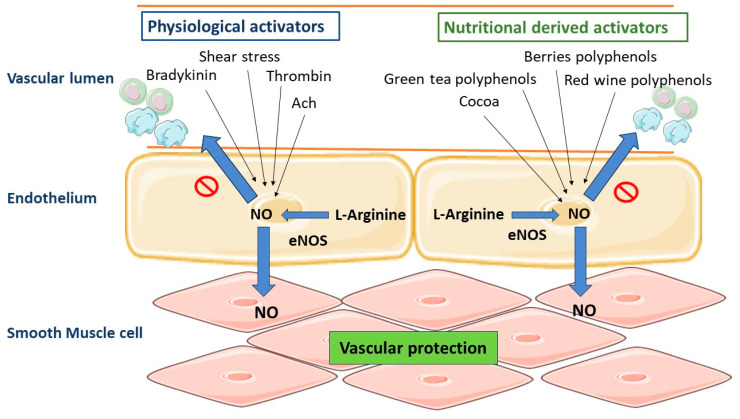
Numerous physiological activators on the endothelium surface can boost endothelial NO production to produce vascular protection. Also, polyphenol-rich dietary items, including chocolate, berries, red wine, and green tea, can enhance endothelial NO production. The endothelial NO synthase (eNOS) enzyme produces NO from L-arginine, exerting a vasodilatory effect. NO can easily diffuse into the cells of vascular smooth muscle, where it triggers guanyl cyclase, thus accumulating cyclic guanosine monophosphate (cGMP), which ultimately activates the protein kinase G and causes vasorelaxation in endothelial.

Diets like the Mediterranean diet have been linked to better cardiovascular health [121], which might be due to the high consumption of polyphenol-rich drinks and foods, as well as fruits and vegetables. Polyphenol-rich foods, including red wine, chocolate, green tea, and berries, also help to promote cardiovascular health [122,123]. Polyphenols have been linked to improving cardiovascular health in various ways. Their advantages include an improvement in lipid profiles. They also have direct effects on endothelial cells and have anti-atherosclerotic, anti-hypertensive, and anti-inflammatory properties (Figure 8).

## 6. Pathophysiology: Oxidative Stress and CVD

In healthy cells, antioxidant defense systems such as superoxide dismutase (SOD), catalase (CAT), and glutathione reductase (GSR) limit the generation of radicals during various physiological activities such as metabolism and cellular respiration [1,124,125]. Long-term exposure to stress [126], pollution [127], smoking, and excessive drinking [128,129], as well as aging [130], can cause an imbalance of oxidative species (also known as reactive oxygen species; ROS) in comparison to endogenous defenses, resulting in oxidative stress [1,124]. ROS can bind to proteins, lipids, and DNA, thus oxidizing them and changing a healthy state into a diseased one. An increase in the level of ROS results in oxidative stress, and the cell’s antioxidant system may become overburdened, endangering the health and integrity of the cell [131].

A similar pathological mechanism, atherosclerosis, underpins cardiovascular illnesses such as coronary artery disease, ischemic stroke, and peripheral artery disease [132]. Atherosclerosis is a multifactorial, degenerative ailment of the medium and great conduit arteries that is fueled by lipid buildup in the artery wall [133,134]. The risk factors of this disease include old age, chronic smoking, hyperlipidemia, hypertension, and a history of diabetes. The atherogenic process is tightly linked to inflammation and endothelial dysfunction [132,135]. Endothelial damage due to ROS leads to the development of atherosclerosis, which may result in myocardial infarction and ischemic reperfusion [136,137,138]. Oxidative stress and ROS target all body cells, especially smooth muscle cells and endothelial cells, with the help of neutrophils, macrophages, and platelets [139].

ROS has an impact on a variety of endothelium-related processes [2]. The most well-known is endothelium-dependent vasorelaxation, which has long been marked as a key component in the prognosis of cardiovascular health, which is harmed by a decrease in NO bioactivity and/or bioavailability [140,141]. There are some mechanisms responsible for reduced NO bioavailability. This can either be caused by a decreased expression of the enzyme responsible for the NO production in endothelial cells, i.e., endothelial NOS (eNOS), or a decrease in the existing NO owing to ROS destruction, among other things [141]. NO is a powerful vasodilator that also inhibits the activation and adherence of inflammatory cells [142]. 

In the vasculature, there are several sources of ROS, including mitochondrial enzymes such as NADH/NADPH oxidase and xanthine oxidase [141,143]. Endothelium-derived NO reacts quickly with the superoxide radical (O_2_^−^) to create peroxynitrite (ONOO), a potent oxidant that is equally damaging to endothelial cells [144]. In this respect, studies have shown that the continued contact of endothelial cells with oxygen and different oxidants like hydrogen peroxide, ONOO, and/or oxidized LDL (ox-LDL) causes epithelial damage by promoting apoptosis, leading to cell damage and endothelial cell dysfunction, which has been reported as a critical early step in atherogenesis. Atherosclerotic lesions can arise from leaky and dysfunctional endothelium [132,133].

LDL normally diffuses easily in both directions across the compromised endothelium. Oxidative stress converts LDL to ox-LDL by peroxidation, which has cytotoxic effects and can cause inflammation [145]. The first step in the formation of atherosclerotic plaques is the oxidation of LDL and its subsequent passage through the endothelial barrier. Furthermore, the interaction of hypercholesterolemia, oxidative stress radicals, and inflammatory molecules creates an environment conducive to severe endothelial damage, which is a characteristic of atherosclerosis development [124,146]. 

A particular adhesion molecule called VCAM-1, which is crucial for binding monocytes and T cells before they transmigrate into the arterial wall, is produced by wounded or activated endothelial cells [145]. Reportedly VCAM-1, ICAM-1, and E-selectin enhance the adherence of leukocytes to the vascular endothelium at the sites of atherosclerotic lesions, consequently boosting signal transduction cascades [146]. These monocytes develop into macrophages after activation, which then become puffy with the uptake of ox LDL by scavenger receptor-mediated phagocytosis, resulting in fatty bands in the artery wall [132,140,147]. Furthermore, lipid-engorged macrophages (foam cells) eventually die in situ because of necrotic cell death, resulting in the creation of a tender and unstable core inside the atherosclerotic plaques, which has a high consistency of lipids [148]. This plaque is stabilized by a protective cap secreted by smooth muscle cells. It consists of a collagen-rich matrix comprising fibroblasts, which can stop the disease from progressing. Prolonged inflammation, on the other hand, might result in plaques that are unstable and prone to rupture [1,148]. Such ruptured plaques cause a fast thrombotic reaction, resulting in arterial blockage and, depending on the location of the atherosclerotic lesion, potentially causing heart attacks, ischemic strokes, or peripheral ischemia [133]. Research findings have linked the plaques in the walls of coronary arteries to chronic atherosclerotic lesions, which can limit the channel lumen. The current preferred hypothesis is that acute coronary syndrome (ACS) is caused by a rupture of the fibrous cap of an atherosclerotic plaque, which facilitates blood contact with extracellular matrix collagen and tissue factors previously deposited in the plaque, resulting in the formation of a thrombus [149,150,151,152]. On the other hand, increased platelet activation, including the adhesion, secretion, and aggregation at the site of arterial injury or in atherosclerotic arteries, plays a key part in the etiology of CVD [153,154]. When an atherosclerotic plaque ruptures, activated platelets can bind to the endothelium, causing fastening, aggregation, and thrombus development, which leads to embolism and the constriction of vessels, two features of myocardial infarction [155].

Physiological hemostasis is a natural defense against excessive blood loss that is based on the creation of a regulated thrombus at the site of the blood vessel injury. Platelets, the smallest (2–4 m) blood corpuscles, are formed at a rate of 40 × 10^3^/mL/day in the bone marrow from megakaryocytes and play a key role in hemostasis. Platelets play a function in hemostasis that extends beyond forming the platelet plug, which also serves as the site of fibrin production (at the site of the vessel wall injury), to include a beneficial influence on vessel wall contraction and participation in clotting responses [8]. Collagen and tissue factor (TF) located in the sub-endothelial matrix come into contact with the lowing blood when the endothelium is injured, causing a clot to develop [156]. Denuded collagen directly promotes platelet pooling and activation, and the denuded tissue factor starts the synthesis of thrombin, which not only transforms fibrinogen to fibrin but also activates platelets [150]. The presence of collagen receptors (including integrin a2b1 and glycoprotein complex GPIb/IX/V) on the platelet surface allows platelets to connect with the subendothelial layer. Platelet attachment leads them to change shape from discoid to spherical, resulting in the creation of pseudopodia and the release of chemicals held in granules (e.g., ADP, P-selectin, von Willebrand factor [vWF], thrombospondin) and, as a result, platelet aggregation [149,157].

## 7. Beneficial Effects of Polyphenols on Cardiovascular Disorders

### 7.1. Polyphenols as Antioxidant Therapy

Antioxidant therapy is becoming better recognized as a strategy for reducing ROS in the vasculature and, as a result, reducing their harmful effects [158]. Blockers of the angiotensin-converting enzyme (ACE) that lower circulatory Ang II detoxify in addition to exhibiting their antihypertensive attributes. In this regard, statins (cholesterol-lowering drugs) are used for the same intent in addition to their cholesterol-lowering properties by regulating HMG CoA reductase. Similarly, vitamins E and C are widely used as dietary supplements in combination with other drugs to reduce oxidative stress [158]. Polyphenols, on the other hand, are gaining attention as possible therapeutic agents for reducing oxidative stress and thereby protecting people from heart diseases [159,160]. In the diet, polyphenols are the most prevalent antioxidants, and their consumption is ten times that of water-soluble vitamin C and one hundred times that of lipid-soluble vitamin E and carotenoids [15]. 

Polyphenols have enormous antioxidant features. The presence of catechol groups, as well as hydroxylation patterns, such as the 3-hydroxy group in flavanols or electron shortage in anthocyanins, is essential in the antioxidant actions [161,162]. The presence of a catechol ring in the structure of various polyphenols has been positively linked with their antioxidant activity, as demonstrated by the ferric-reducing ability power (FRAP). In one study, further increases in FRAP were achieved by using aliphatic substitution or a double bond in the aliphatic group conjugated with the catechol ring; there were no benefits from adding more OH groups [163]. Polyphenols may serve as antioxidants by scavenging free radicals in a variety of ways. They exert their antioxidant potential either by inhibiting or potentiating the activity of various enzymes or by direct interaction with free radicals [164]. ROS that can be extremely toxic to lipids, proteins, and DNA include superoxide (O_2_^−^), hydrogen peroxide (H_2_O_2_) [165], and hypochlorous acid (HOCl) [166], which are all immediately scavenged by polyphenols like quercetin and catechin. In this respect, ROS can be made less reactive by the phenolic core acting as a buffer and collecting electrons [167]. Polyphenols can have indirect effects on cellular detoxification systems such as catalase (CAT), superoxide dismutases (SODs), and glutathione peroxidases [168,169]. Polyphenols can also inhibit enzymes that produce ROS, such as xanthine oxidase and nicotinamide adenine dinucleotide phosphate (NADPH) oxidase [170,171]. In addition to the production of ROS, there is an increase in the quantities of free metal ions. Due to their low redox potentials, flavonoids can chelate these metal ions, which prevents the production of free radicals. Research findings have indicated that quercetin is the flavonoid with the best capacity to chelate metal ions [171,172,173]. 

Polyphenols are known to be potent antioxidants due to their health advantages. Phenolic compounds may easily donate an electron or H atom from an aromatic hydroxyl group to a free radical, effectively neutralizing its effects. It all comes down to how the functional groups are arranged in the polyphenol’s core structure [174]. Although polyphenols have been shown to have good antioxidant activity in vitro, their antioxidant capacity in vivo is lower than it is in vitro. Several variables play a role, including the metabolism of polyphenols into compounds with lower antioxidant activity. By inhibiting the –OH group, metabolism reduces polyphenols’ ability to scavenge radicals [1,175]. Because proteins, uric acid, vitamin C, and thiols create an antioxidant barrier strong enough to overlook phenolic contribution in plasma, the polyphenolic antioxidant contribution is low [176]. Taking this into account, the theory that eating polyphenol-rich foods boosts plasma antioxidant capacity is debunked. Other dietary components absorbed alongside polyphenols, such as vitamins C and E, may be to blame for this increase [177]. The recognized interaction between fructose and uric acid is more likely to cause the antioxidant effect of a fruit- and vegetable-rich diet [178].

### 7.2. Polyphenols and Vascular Tone

The significance of endothelium-produced nitric oxide (NO) in controlling vascular tone and blood pressure is well understood. The central mechanism of NO action is the activation of the cGMP-protein kinase G cascade in artery smooth muscle cells. The potassium channels are triggered when the cascade is activated, resulting in membrane hyperpolarization and preventing intracellular calcium influx, which induces vasodilation. On the other hand, protein kinase G reduces smooth muscle vasoconstriction in arteries by phosphorylating myosin light chains [179,180]. NO generation is primarily responsible for the polyphenols’ effect on the endothelium [181,182,183].

After ingesting red wine or polyphenols (1 g/kg body weight) circulating NO concentrations reach 30 and 40 nM after 30 min in adults. A decrease in blood pressure (11 mmHg) and an increase in heart rate have also been observed [184]. Research findings have shown that olive oil can help hypertensive people lower their blood pressure [185], whereas red wine polyphenolic compounds (RWPC) can produce the endothelium-dependent relaxation of isolated arteries such as the rat’s mesenteric artery or aorta [181]. In addition, red wine polyphenols, polyphenols from grape skin, and quercetin exhibit antihypertensive effects. In this respect, short-term oral treatment with RWPC lowers blood pressure in normotensive rats. This hemodynamic effect was correlated with enhanced endothelium-dependent relaxation and the induction of the genes responsible for inducible NO synthase and COX-2 inside the artery wall, thus contributing to the maintenance of agonist-induced contractility [186]. The higher synthesis of NO in consequence to the impact of polyphenols found in wine extract is linked to the calcium ion-dependent pathway, among several other things [187]. Resveratrol and quercetin cause an increase in the intracellular ion concentration of (Ca^2+^) ions through the opening of potassium channels or the inhibition of Ca^2+^ ATP-ase within the endoplasmic reticulum of endothelial cells [188,189]. Similarly, delphinidin, an anthocyanin present in natural foods like red wine, can activate endothelial cells. This anthocyanin raises intracellular protein-Ca^2+^ and tyrosin phosphorylation, which controls eNOS. Tyrosine kinases and phospholipase C are both involved in Ca^2+^ signaling [190]. Furthermore, RWPC might even enhance endothelial NO production via the redox-responsive PI3/Akt channel, according to another report [191]. 

In addition, the effect of polyphenolic compounds on endothelial cells in preventing cardiovascular diseases is not limited to the stimulation of NO production. Because of the increased production of PGI2, the vasodilating effect is also boosted. In vitro studies on human endothelial cells exposed to the action of cocoa extract rich in procyanidins at a concentration of 2 mg/L and in vivo studies on procyanidins contained in chocolate administered to healthy volunteers showed that the ratio of cysteinyl leukotrienes (LTC4, LTD4, LTE4) to PGI2 can be reduced by 58 and 52%, respectively [192]. In contrast, isolavonoids, particularly genistein, limit the procoagulant action of vascular endothelium by, for example, lowering ET-1 expression [193]. Finally, polyphenols can affect endothelial cells’ NO levels by affecting PDE-2 and PDE-4, two phosphodiesterases [194,195]. Taken together, plant polyphenols may have complex effects on the circulatory system’s NO balance, which could account for their antihypertensive effects [196].

### 7.3. Polyphenols and Atherosclerosis

Atherosclerosis is the hardening and narrowing of the arteries, which is triggered by the buildup of lipids, cholesterol, and other substances in and on the artery walls over time. This then progresses into the endothelium, where they are oxidized by endothelial smooth muscle cells and activated macrophages [197,198]. ROS and reactive nitrogen species (RNS) production can enhance LDL oxidation. This causes a buildup of macrophages in this area, which clear oxidized LDL and transform them into foam cells. Endothelial dysfunction, as well as the concentration of monocytes/macrophages in the vascular intima under the influence of chemokines and adhesion molecules, foam cell development, and vascular smooth muscle proliferation, are all linked to the inflammatory backdrop of atherosclerotic lesions [199]. There is also a rise in extracellular matrix buildup surrounding the spot of inflammation, leading to plaque development which blocks the vessel, resulting in the loss of the blood artery’s natural capacity to relax [198,199].

Published research has dealt with the potential benefits of polyphenols on atherosclerosis. Within this context, various studies have shown that RWPC and purple grape juice slow down atherosclerosis onset and progression through their anti-LDL oxidation, antioxidant properties, and inhibition of platelet aggregation properties, as well as an increase in HDL concentration and delay of vascular smooth muscle cell (SMC) propagation. In conclusion, polyphenols may be able to maintain “healthy blood vessels” by producing NO, which is important for vascular tone [200,201,202]. Recently, researchers found that giving rabbits red wine polyphenolic compounds (RWPC) orally reduces neointimal growth, lipid buildup, and inflammation in their iliac arteries. This is because RWPC has an anti-inflammatory effect [203]. In addition, when hamsters are given red wine, they have less neointimal hyperplasia, which is caused by a decrease in a protein that helps monocytes enter the artery wall. This is one of the ways that the artery reopens [204].

### 7.4. Polyphenols and Anti-Platelet Action

The excessive activation of platelets is linked to several long-term vascular diseases. This is due to the many adhesion proteins in the granules that, when highly activated, can lead to different types of thrombotic diseases [205,206]. In this respect, numerous important things happen in the process of platelet activation. One of them is the conversion of arachidonic acid to thromboxane A2, an arachidonate metabolite, through the cyclooxygenase pathway [207]. In this context, polyphenols are valuable from the perspective of platelet activation, which includes the adhesion and aggregation of platelets, due to the antioxidant effect of polyphenols. The first step in platelet activation involves platelets sticking to the collagen in the body; as a result, the platelets become activated. In this respect, proteins like fibrinogen and thrombospondin act as adhesion proteins, and platelet receptors work together to help platelets stick together, leading to the start of a signaling process inside cells and the activation of platelets [208]. However, it has not been fully explained how polyphenols make platelets less likely to stick together. It turns out that extracts rich in polyphenolic compounds, like grape seed and Yucca schidigera extracts, can help stop platelets from sticking to collagen. These extracts contain resveratrol and its derivatives, which make platelets less likely to stick together when they are stimulated by thrombin [153]. 

Thromboxane A2 (TXA2) is the key compound that is formed from the breakdown of arachidonic acid (ARA). It has some surface receptors that make platelets clump together. Evidence from the literature has indicated that the anti-aggregative effect of polyphenols is linked to numerous complicated molecular processes [209]. The capacity of polyphenols to hinder the enzymes involved in the formation of TXA2, COX, and LOX is the primary method by which they exert their anti-platelet aggregate effects on platelets [209,210]. However, they are also antagonists of the thromboxane A2 receptor, which suggests that flavonoids, through their indirectly suppressive effect on COX1, can lower TXA2 levels in the blood [211]. In an in vivo dog model, researchers investigated the effects of grape juice and red and white wine on platelet aggregation activity. The results revealed the antiplatelet effects of red wine and grape juice, while white wine does not yet have this impact [212]. 

Flavonoids have been shown to lower platelet aggregation because collagen metabolism is altered by these compounds, in addition to their interference in arachidonic acid metabolism. This is expressed as the antiplatelet action of collagen in the early stages of the aggregation of platelets. Moreover, the oxidative stress results in the aggregation of platelet in response to collagen via the activation of the inositol pathway, boosting intracellular calcium levels in the process. Flavonoids such as quercetin, catechin, and kaempferol, among others, have been shown to decrease oxidative stress by impeding the enzyme NADPH-oxidase [205].

### 7.5. Polyphenols as Anti-Inflammatory Agents

Inflammatory response to injury is a complicated biotic process that happens in response to a damaging stimulus. Different enzymes, including cyclooxygenase (COX), lipoxygenase (LOX), tyrosine kinase (TK), phospholipase A2 (PLA2s), and protein kinase C), are responsible for the proper function of an inflammatory response. Certain flavonoids have been demonstrated to act directly on several such enzymes, blocking them and therefore directly affecting inflammation [213,214]. One of the most important elements in preventing and treating chronic inflammation, according to epidemiological research, is nutrition. Through ex vivo and in vivo models, researchers have discovered that some flavonoids exert anti-inflammatory effects. One of the key bodily functions that flavonoids have an impact on is the synthesis of prostaglandins. Hesperidin and diosmin can reduce the generation of prostaglandins, according to several in vivo studies [215].

The mobilization of leukocytes is known as a critical stage in the progression of inflammation that occurs in cardiovascular illnesses and other conditions. The production of arachidonic acid ultimately results in the generation of cytokines (IL-1) and chemokines (IL-8) by neutrophils, which is mediated by both COX and LOX. In this regard, quercetin, a polyphenol, is especially effective in suppressing the formation of prostaglandins (PGs), leukotrienes (LT), and thromboxanes (TXA) by preventing the enzymes COX and LOX, respectively [216,217,218]. Evidence from numerous ex vivo experiments shows that some flavonoids, for example, bilobetine, morelloflavone, amentoflavone, and those found in *Sophora flavescens*, exert their effect by inhibiting the production of arachidonic acid [219]. Furthermore, resveratrol is regarded as a molecule with anti-inflammatory properties, as it inhibits the production of PGs [220]. Table 3 lists the key cardioprotective mechanisms of action behind the beneficial effects dietary polyphenols have on human health.

## 8. Interactions between Polyphenols and Nutrients and Drugs

Flavonoids form protein complexes through several nonspecific ways, including hydrogen bonds, hydrophobic interactions, and covalent bonds [221,222,223]. By forming complexes with proteins, polyphenols can alter their function, structure, solubility, hydrophobicity, thermal stability, isoelectric point, and susceptibility to digestive enzymes [223]. These modifications can influence digestion and the utilization of dietary proteins. In addition, polyphenols may affect the structure of digestive enzymes such as amylases, proteases, and lipases, disrupting their function and causing biochemical processes to malfunction. Furthermore, polyphenols can modify the processes of drug absorption, distribution, and metabolism. This is achieved via two mechanisms: the inhibition of P450 activity, which can occur through competitive, non-competitive, or uncompetitive enzyme inhibition, and the reduction of P450 activity. These alterations in P450 activity directly impact the clinical outcomes of drugs [221].

Similarly, polyphenols interact with reactive oxygen species, free radicals, and many other chemical compounds within their immediate environment due to their high concentration of active functional groups. In some cases, specific interactions can have adverse effects on human health [224]. It is critical to consider the interaction between polyphenols and pharmacological agents, namely the iron-containing preparations used to treat anemia. This interaction can significantly affect drug metabolism and pharmacokinetics and may modify pharmaceutical therapeutic effects [225]. For example, consumers acknowledge that grapefruit juice and herbal infusions are contraindicated for consuming pharmaceutical medications concurrently. These modifications can cause an increase in treatment effectiveness or a decrease in effectiveness. Furthermore, polyphenols have been demonstrated to have a significant effect on the drug-metabolizing enzymes involved in phases I and II of drug metabolism, although the fundamental rationale for such recommendations remains undisclosed; these enzymes include cytochrome P450, glutathione S-transferase, UDP-glucuronosyltransferase, sulfotransferase, N-acetyltransferase, methyltransferase, epoxide hydrolase, and NAD(P)H, a quinone oxidase transporter [223,224,225].

## 9. Adverse Effects of Polyphenols

The human diet contains a vast array of polyphenolic chemicals, about 8000 [226]. However, it has been found that consuming excessive amounts of polyphenols can lead to side effects [227]. In this regard, green tea extracts that include the well-known catechin (−)-epigallocatechin-3-gallate (EGCG) are marketed with the intention of facilitating weight loss; however, hepatotoxicity has been reported in a subset of individuals who consumed the product [228]. Similarly, chlorogenic acid, a prevalent polyphenol found in coffee, has been associated with numerous health advantages, as well as cytotoxic and genotoxic effects [229]. Polyphenols have been found to elicit mutagenic effects, promote the development of cancer, and produce genotoxicity. Within this context, multiple investigations have demonstrated that flavonoids engage in interactions with topoisomerase II and II, resulting in the induction of DNA cleavage. The present paper provides evidence that genistein exhibits an augmenting effect on the DNA cleaving activity of both human topoisomerase II and II, as reported in a previous study [230]. According to prior studies, it has been observed that (−)-epigallocatechin gallate (EGCG) [231] exhibits redox-dependent characteristics as a topoisomerase II toxin through the creation of covalent linkages with the enzyme. Moreover, numerous studies have shown that polyphenols exert inhibitory effects on topoisomerase via multiple pathways. Research findings have indicated that topoisomerase II poisons have an impact on EGCG, a compound that is influenced by redox reactions. The toxins belonging to the kaempferol and quercetin classes have mechanisms of action that are dependent on redox processes, as well as additional action mechanisms. Research findings have shown that the effects of (−)-epicatechin gallate (ECG) and (−)-epicatechin/EC/ are statistically insignificant [232].

Although polyphenols exhibit potent antioxidant effects, they may demonstrate prooxidant characteristics in the presence of elevated amounts of metals, pH, and oxygen. Specifically, copper and iron contribute to the heightened prooxidant activity of EGCG. The chemical exhibits prooxidant characteristics through the formation of a redox complex with either a transition metal ion or a phenoxyl radical [233]. Phenoxyl radicals produce reactive oxygen species (ROS) such as O_2_• and H_2_O_2_ in response to the presence of oxygen. Consequently, these chemical substances induce DNA damage, lipid peroxidation, and various signs of molecular oxidation. It has been proposed that the potential pro-oxidant impacts of polyphenols, specifically regarding EGCG, elicit noteworthy responses [234]. Previous studies have noted that the oxidation of polyphenols possessing small molecular structures, such as dihydroxycinnamic acids, leads to DNA incision or lipid peroxidation [233]. Zeng et al. [235] conducted a study that revealed that compounds possessing dihydroxyl groups in the ortho-conformation, such as caffeic acid and chlorogenic acid, as well as those containing 4-hydroxy-3-methoxyl groups, such as sinapic acid and ferulic acid, induced a significantly higher level of DNA damage compared to compounds lacking these functional groups. 

## 10. Materials and Methods

### 10.1. Literature Search and Methodology

In this current review on Plant polyphenols and their potential benefits on cardiovascular health, different databases, including PubMed, Google Scholar, NIH National Library of Medicine, Scopus, and Web of Sciences were surveyed to retrieve data using a series of search terms, namely “cardiovascular system”, “Coronary artery disease”, “Polyphenols”, “vascular Homeostasis”, “Vascular endothelium”, “Oxidative stress”, “polyphenols as antioxidants”, and “Atherosclerosis”, without any publication date limit. Research articles and reviews were included, whereas conference abstracts and non-English publications were excluded. 

### 10.2. Illustrations and Figures

The chemical assemblies were drawn using ChemDraw 22.0.0 with the assistance of PubChem. The figures demonstrating mechanisms of action were drawn in Microsoft PowerPoint 2019 and Biorender (https://biorender.com/, accessed on 25 May 2023).

## 11. Conclusions

Food and plant-derived natural substances are attractive because they are relatively safe compared to synthetic drugs, affordable, and widely accessible. At present, people consume natural food products obtained from fruits and vegetables and other food items to treat and manage cardiovascular diseases. These food items contain natural compounds, especially polyphenols, which exhibit antioxidant properties among other things. There is uncertainty regarding the precise physiological actions of these substances, particularly regarding their effects on the cardiovascular system. In summary, the findings presented in this work show that significant amounts of polyphenols can be found in fruits, especially berries, and drinks such as tea and coffee. Similarly, substantial amounts of these potential cardioprotective compounds are present in vegetables, leguminous plants, and grains. In addition, the findings presented in this paper demonstrate the anti-atherosclerotic properties of dietary polyphenols, including improvements in endothelium and vascular function, hemostasis and platelet function, and inflammatory biomarkers. Long-term dietary intervention research comparing the effectiveness of various dosages of conventional pharmaceuticals to polyphenol treatments in a range of clinical populations will increase the clinical acceptance of polyphenol therapies. The scientific community would benefit greatly from more research into the mechanisms underlying in vivo bioavailability in humans and the safety implications of consuming foods high in polyphenols. Although there have been some debates over its absorption, oral polyphenol consumption has shown encouraging results as a supplemental treatment option in lowering atherosclerosis development in at-risk patients. However, more extensive studies involving human subjects are required to establish the efficacy and safety of dietary polyphenols for long-term use in treating human diseases such as cancer and cardiovascular diseases. 

There are numerous mechanisms through which polyphenols can influence the complex pathophysiology of cardiovascular disease (CVD); these include decreasing blood pressure, reducing cholesterol levels, acting as antioxidants, mitigating inflammation, inhibiting cell proliferation and angiogenesis, promoting endothelial function recovery, preventing thrombosis, and providing protection for the myocardium, among other functions. Nevertheless, there are several significant challenges that prevent the clinical utilization of polyphenols. These include dosage, specificity, potency, practicality, and the potential short- or long-term side effects on human subjects. Although natural polyphenols are generally considered to be safe, their potential adverse effects depend on their distribution and target cells within the body. It is possible for certain polyphenols to interact with conventional drugs as well as nutrition, thereby posing potential safety risks. Future animal experiments, large-scale cohort studies, and human intervention trials are required to address these challenges.

## Figures and Tables

**Table 1:** Polyphenol-rich plant foods.

Plant Food	Latin Name	Edible Part	Concentration mg/100 g	Major Polyphenols	References
Apple	*Malus domestica*	Peel	50–120 ^y^	Phlorizin, quercetin, phenolic acids (chlorogenic acid)	[23,24]
		Flesh	0.2–0.9		
		Total	5–50		
Blackberry	*Rubus fruticosus*	Whole	130–405	Anthocyanins, flavanols (EC), phenolic acid (ellagic acid)	[25]
Blueberry	*Vaccinium corymbosum*	Whole	160–480	Anthocyanins, flavonols (quercetin), phenolic acids (chlorogenic acid)	[25]
Coffee	*Coffea arabica*	Beverage, filtered	90	Phenolic acids (chlorogenic acid)	[25]
Chestnut (raw)	*Castanea sativa*	Whole nut	547–1960	Hydroxybenzoic acids (gallic acid, ellagic acid), tannins	[25]
Cacao	*Theabroma cacao*	Beans, powder	300–1100 ^x^	Flavanols (EC)	[25]
Green tea	*Camellia sinensis*	Extract	29–103 ^x^	Flavanols (EC, EGCG)	[25]
Grapefruit	*Citrus x paradisi*	Flesh	15–115	Flavonoids, phenolic acids	[25]
Olive oil, extra virgin	*Olea europaea*	Whole oil	4–200	Tyrosols, lignans (pinoresinol), phenolic acids, hydrolyzable tannins	[25]
Potato	*Solanum tuberosum*	Peel	180–5000	Phenolic acids (chlorogenic acid)	[25,26]
		Flesh	1–1000		
		Total	10–50		
Plum	*Prunus domestica*	Total	130–240	Phenolic acids (chlorogenic acid), procyanidins, anthocyanins	[25]
Pomegranate	*Punica granatum*	Juice	240 ^x^	Punicalagin (and ellagitannin)	[27]
Grapes, Red wine	*Vitis vinifera*	Final product	25–300 ^x^	Phenolic acids, anthocyanins, tannins, stilbenes (resveratrol)	[25]
Wheat	*Triticum aestivum*	Whole grain	85–220	Phenolic acids (hydroxybenzoic acids, hydroxycinnamic acids)	[25]
Spinach	*Spinacia oleracea*	Leaf	30–290	Flavonols	[25]

Abbreviations: EC = epicatechin; EGCG = epigallocatechin gallate. ^x^ = In juices, wine, and other beverages: mg/100 mL. ^y^ = Concentration in mg/cm^2^. Note that the polyphenol content in purple potatoes is approximately five times higher than that in other varieties.

**Table 2:** The most prevalent dietary polyphenols and their major colonic metabolites.

Polyphenol Class	Metabolites	Bioavailability	References
**Anthocyanins**	Catechol Glucunoride conjugates Hydroxyhippuric acid Methyl conjugates Propionic acid Protocatechuic acid Pyrogallol Sulphate conjugates Syringic acid Vanillic acid	**Absorption:** A minor amount of glycosylated anthocyanin product is immediately absorbed in the gut, resulting in maximal plasma concentrations ranging from 14 to 592 nmol/L at 4–5 h after intake (doses: 68–1300 mg). **Metabolism:** Through glucosidase activity, the gut bacteria hydrolyze anthocyanins. Cleavage of the C3-ring breaks down the aglycones, which are then metabolized into various phenolic and aldehydic components. **Excretion:** Urinary excretion is estimated to be between 0.03% and 4% of the ingested dosage, with elimination half-lives of 15–3 h.	[92,100,101,102,103]
**Phenolic acids**	Dihydrocaffeic acid Feruloylglycine Dihydrofeluric acid Hydroxybenzoic acid Vanillic acid Hippuric acid	**Absorption:** Approximately 30 min after consumption, the maximal plasma concentration level is attained, and this is because its maximum absorption occurs in the small intestine. **Metabolism:** These chemicals are metabolized and circulated in the body as glucuronate, sulfate, and methylated metabolites with varying degrees of bioactivity. **Excretion:** Urinary excretions account for roughly 40% of total consumption, with excretion peaking after 8 h.	[92,100,104,105]
**Flavonols**	Hydroxyphenylacetic derivates Protocatechuric acid Propionic acid	**Absorption:** Small intestine absorption is poor. **Metabolism:** The flavonol skeleton is broken down by gut microbiota microbial enzymes, resulting in the production of low-molecular-weight polar metabolites. **Excretion:** The clearance of epicatechin metabolites relies heavily on urine excretion.	[92,100,106]
**Flavan-3-ols and** **Proanthocyanidins**	Benzoic acids Hippuric acids Phenilvalerolactones Phenylacetic acids Phenylpropionic acids Phenylvaleric acids	**Absorption:** The small intestine absorbs between 8 and 17 percent of monomeric-3-ols. **Metabolism:** The leftover unabsorbed portion reaches the other end of the large intestine practically intact, and their gut bacteria cause the breakdown of the flavonoid skeleton, producing several low-molecular-mass metabolites.	[19,92,100,107]
**Ellagitanninis**	Dimethyl-ellagic acid Urolithin A and B Urolithin D	**Absorption:** Ellagitannins are hydrolyzed in the gastrointestinal lumen after intake, yielding a free form of ellagic acid. **Metabolism:** The gut microbiota degrades ellagic acid in the large intestine, resulting in a variety of derivative chemicals known as urolithins, all of which have the same nucleus. Urolithins are substantially absorbed and metabolized as glucuronidated and sulfated products by hepatic and intestinal cells.	[92,108,109]
**Stilbenes**	3,4′-dihydro-trans-stilbene Dihydroresveratrol 3,4′-dihydroxybibenzyl	**Absorption:** The upper gastrointestinal tract absorbs resveratrol. **Metabolism:** Enterocytes and hepatocytes both metabolize it, producing glucuronide and sulfate forms.	[92,100,110,111,112,113,114]

**Table 3:** Cardioprotective mechanisms of action of polyphenols.

Beneficial Effect	Specific Mechanism
**Antioxidant**	Generation of stable flavonoid radicals, increasing the protection of antioxidant systems and the elimination of ROS [83,84,85,86,87,88,89,90,91,92,93,94,95,96,97,98,99,100,101,102,103].
**Antihypertensive**	Modulate the RAAS and prompt an increase in the endothelium-derived nitric oxide concentration [125,126,127,128,129,130,131,132,133,134,135,136,137,138,139,140,141,142,143].
**Anti-atherogenic**	Through their antioxidant action, the diminished oxidation of LDL, and antiplatelet clumping action, they inhibit the formation and progression of atherosclerosis. They also, inhibit the oxidative degradation of lipoproteins and decrease the circulatory lipid levels [123,124,125,126,127,128,129].
**Antiplatelet**	Inhibitory effect on excessive platelet activation and produce decreased platelet adhesion [130,131,132,133,134,135,136,137].
**Anti-inflammatory**	Blocking inflammatory enzymes (COX, LOX, TK, PLA2s, protein kinase C), interfering with the production of prostaglandins, and suppressing the formation of PGs, LT, and TXA [159,160,161,162,163,164,165,166].

## Data Availability

Not applicable.
